# Using patient comments from a standardised experience survey to investigate their perceptions and prioritise improvement actions: a thematic and syntactic analysis

**DOI:** 10.1186/s12913-023-09953-z

**Published:** 2023-09-14

**Authors:** Marion Crubezy, Céline Douay, Philippe Michel, Julie Haesebaert

**Affiliations:** 1 Research On Healthcare Performance (RESHAPE), INSERM U1290, Université Claude Bernard Lyon 1, Lyon, France; 2 Institut d’études KPAM, Paris, France; 3 Direction Qualité Usagers Et Santé Populationnelle, Hospices Civils de Lyon, Lyon, France; 4 Pôle de Santé Publique, Service de Recherche Et Epidémiologie Cliniques, Hospices Civils de Lyon, Lyon, France

**Keywords:** Patient Experience, Patient-reported Experience Measures, Healthcare quality improvement, National patient experience surveys, Analysis of comments

## Abstract

**Background:**

Although patient experience surveys flourish in many countries with the aim to improve quality of care, questions remain concerning their ability to become effective drivers of change within institutions. The patient comments from the French national patient experience hospital survey were analysed using an innovative structured approach to characterise patient experience and identify field actions for the institutions.

**Methods:**

The comments were taken from the two open-ended questions comprised in the patient experience survey of the *Hospices Civils de Lyon* between 2018 and 2019. The comments analysis methodology consisted in three steps: thematic analysis; syntactic analysis; generation of statistics for the creation of a patient journey and prioritisation of sub-themes. The STROBE statement checklist was followed.

**Results:**

Over a year, 79.7% of the 7 362 respondents left at least one comment at the end of the survey and were included in the study, for a total of 5 868 surveys and 10 061 comments. These led to the identification of 28 general themes and 184 specific sub-themes. From the patient journey created, 23 sub-themes were prioritised and gathered into four key categories: relationship between patient and staff; environment; surgery and pain management; information and care coordination. For each of them, the actions and expectations formulated by the respondents were described.

**Conclusions:**

The analysis of patient comments obtained from a standardised survey allowed to characterise the patient journey using data that describes patient experience, enabling a prioritisation of actions aiming to improve practice and quality of care at the institution, department, and staff level.

**Supplementary Information:**

The online version contains supplementary material available at 10.1186/s12913-023-09953-z.

## Background

Patient Experience (PE) has been recognised as a useful measure to help evaluate and improve healthcare systems [1, 2]. PE measurement and evaluation programmes derived from standardised tools and specifically designed for healthcare institutions have been nationally implemented in several countries [3, 4]. However, patient associations raised two main limitations regarding these programmes [5, 6]. First, it is not certain that the data and evidence generated really reflect the experience of the patients as the approach privileges the evaluation of service quality rather than focusing on all the aspects of PE. Secondly, these programmes do not appear to be effective drivers of change within healthcare institutions. The most common barrier to the use of the results from local or standardised surveys is the difficulty to engage staff as the quantitative results are perceived as not sufficiently specific and are hard to translate into effective interventions [7, 8]. To improve healthcare practices and quality of care, healthcare institutions are increasingly using patient feedback as a tool to inform strategies for improving care, both at the hospital governance, department, and staff levels [9, 10]. For example, a survey showed that more than 40% of outpatient healthcare providers use patient feedback for implementing measures to improve patient care [11]. In this context, we designed an innovative approach to analyse patient comments from the French national patient survey of an academic hospital federation. The hypothesis was that these data allow to reflect patient views and identify specific improvements and practices while at the same time valuing the use of a national patient experience survey. The objectives were to (i) not presuppose an a priori framework for patient experience (to be as close as possible to their experience), (ii) to encompass all experiences (by including all comments and keeping all the subjects mentioned), (iii) to propose a prioritisation of the subjects based on the most important issues for patients, and (iv) to identify, within the comments, actions and initiatives that could help staff and institutions improve their practice.

## Methods

### Materials

This study reports the analysis of qualitative data from the French national patient satisfaction and experience survey named e-Satis. We analysed the comments from the patients who spent at least two consecutive nights in a medical, surgery, or obstetric department (survey called + 48H MCO) of the *Hospices Civils de Lyon* (HCL), an academic hospital federation that includes 13 healthcare organisations bringing together all medical and surgical disciplines. The online survey is sent automatically by e-mail to all patients (if the latter provided an e-mail address on admission) and the patient, a relative, or both can answer up to 10 weeks after the email is sent. The survey contains 62 closed-ended questions and two open-ended questions at the end of the survey. The comments analysed came from these two open-ended questions, which present a positive and negative duality: "*What do you remember as being positive during your stay?*" and "*What do you remember as being negative during your stay?"* [12]. The analysed surveys were recorded between 01/07/2018 and 01/07/2019.

### Guarantee of anonymity

No traceability is possible between the patient and the data collected, thus preventing any issue of anonymity.

### Study design

The comment analysis methodology used for this study translates qualitative material (comments) into quantifiable data. The methodology consists of three main steps: analysis of the meaning of words: constitution of a dictionary of themes and sub-themes; analysis of syntax: measurement of speech engagement; generation of statistics, creation of the patient journey, and prioritisation of sub-themes [13]. The STROBE statement checklist was followed.

### Dictionary of themes and sub-themes

The comments were decomposed into single words or phrases (a combination of morphemes or words that follow each other and produce an acceptable meaning). The text decomposition was carried out by the linguistic analysis software named Qualitative (Lidia SA, Orléans, France) which produced an exhaustive list of all the unique words or expressions present at least once in the comments. A categorisation was then carried out manually by two researchers in a double-blind way to create a dictionary of themes from the exhaustive list of words or expressions. The categorisation was based on the lexical field and the meaning of the words. Words or expressions referring to the same theme were grouped based on two levels of precision: a general theme and a specific sub-theme. In the event of a discrepancy in the identification of themes, a reconciliation was carried out with the support of a third researcher. Beyond the gathering, when a word or expression included a qualifying adjective or words specifying the respondent’s intention, it was also qualified in a positive or negative way. A researcher reviewed the themes and sub-themes and read the comments associated to check if they were coherent between them, distinct from each other, and if they made sense. A definition of the themes and sub-themes was made to describe their subject. Each sub-theme was characterised by a sentence reflecting the content of the comments and by a short description of the qualitative and quantitative data: comments included and characteristics of the respondents mentioning them. Actions, expectations, and practices that were described by the respondents in their comments were also identified.

### Syntax analysis: measurement of speech engagement

This second part of the analysis focused on the syntax used to express ideas rather than the meaning of the words, which was analysed during the construction of the dictionary of themes and sub-themes. Syntax analysis, which examines the way words are arranged, can be used to assess the speaker's level of engagement in their speech. This linguistic indicator provides insight into the importance that the commentator assigns to his/her ideas. The detailed process for this analysis has been previously published [13]. The linguistic analysis software was used to identify the syntactic combinations for each word and expression that is listed in its lexicon. The software's lexicon includes two types of syntactic classes: a closed class, which encompasses all the syntactic elements that can be exhaustively enumerated (e.g., pronouns, determiners), and an open class, which depends on the specific vocabulary of the domain of study. Once the syntactic combinations were identified, the software assigned a predefined implication index on a scale from one to nine to each word and expression. The calculations were performed on all the comments analysed, including all words and expressions from each theme and sub-theme. The software then calculated the mean level of engagement for all the comments, adjusting the scores for each theme and sub-theme based on this mean. The linguistic analysis software was then used to calculate the share of positive and negative speech in each theme and sub-theme identified during the construction of the dictionary. This allowed for the measurement of positive and negative engagement, with scores normalised to a basis of + 100 for positive and -100 for negative, thus ensuring all results had the same maximum value [13–15].

### Generation of statistics, creation of the patient journey, and prioritisation of sub-themes

For each theme and sub-theme identified, three indicators were calculated by the software: the occurrence of each theme and sub-theme (the percentage of respondents who mentioned this theme); the mean satisfaction which included the number of positive, negative, and neutral comments for each theme and sub-theme; the positive and negative speech engagement.

The themes and sub-themes identified were ordered to chronologically follow the patient’s journey from pre-admission to discharge and follow-up. The patient journey was then modelled using Adobe Illustrator (Adobe, San Jose, USA) and graphic elements that illustrated five indicators: (i) the sentence that summarised the sub-theme; (ii) a grey and blue bubble displaying the occurrence of each theme and sub-theme, respectively; (iii) a yellow man representing the mean satisfaction; (iv) a blue bar representing the positive speech engagement; (v) a red bar representing the negative speech engagement.

The identified sub-themes were prioritised according to four criteria: (i) strengths: sub-themes with a high occurrence and a high level of positive speech engagement and a high level of satisfaction; (ii) priorities: sub-themes with a high occurrence and a potential for progression (negative mean satisfaction and a high level of speech engagement); (iii) good practices: sub-themes with a low occurrence and a high level of positive speech engagement; (iv) weak signals: sub-themes with a low occurrence and a high level of negative speech engagement. The thresholds were not strictly defined to reflect reality by not selecting too many sub-themes that could not be dealt with. This also fits with the constitution of a non-predetermined dictionary, as the number of words and expressions in each sub-theme was not driven to attain a specific threshold of occurrence. Using an inductive approach, sub-themes were gathered into key categories and a summary of the quantitative and qualitative data for each of them was provided: prioritisation; description of the sub-theme; content of the comments; quantitative characteristics on this sub-theme; actions/ practices/ expectations identified in the comments.

## Results

### Characteristics of the surveys analysed

Among the 13 institutions of the HCL, eight had the e-Satis survey and comments available at the time of the study period. Over that period, 134 151 patients were hospitalised in one of the departments of one or more of these institutions. The survey was sent to 30 840 patients and 7 362 completed it. Among these, 5 868 questionnaires had a least one comment: 79.7% of the respondents left a comment. The 5 868 questionnaires were included, for a total of 10 061 comments distributed as follows: 5 401 comments for the positive question and 4 660 for the negative question. On average, comments were 145 characters long (average of 93 characters for the positive question and 145 for the negative one). The characteristics of the respondents to the 5 868 surveys included for analysis are reported in Table 1.

**Table 1:** Characteristics of the respondents to the e-Satis survey containing at least one comment received by the HCL between 01/07/2018 and 01/07/2019

Characteristics	*N* = 5 868
**Respondent**	
Patient	5058 (86.2%)
Parent or relative	536 (9.1%)
Both	274 (4.7%)
**Sex**	
Woman	3694 (63%)
Man	2174 (37%)
**Age (yrs)**	
+ 90	53 (0.9%)
80 to 89	269 (4.6%)
70 to 79	865 (14.7%)
60 to 69	1047 (17.8%)
50 to 59	789 (13.4%)
40 to 49	654 (11.1%)
30 to 39	1391 (23.7%)
20 to 29	419 (7.1%)
10 to 19	182 (3.1%)
- 10	199 (3.4%)
**Type of room**	
Single	3557 (60.6%)
Shared	2311 (39.4%)
**Referral**	
Emergency	811 (13.8%)
Physician	2664 (45.4%)
Relative	762 (13%)
Other institution	507 (8.6%)
Other	1124 (19.2%)
**Departments**	
Surgery	2387 (40.7%)
Medical	1916 (32.7%)
Intensive care	31 (0.5%)
Obstetrics/Maternity	1398 (23.8%)
Paediatrics	136 (2.3%)

Results are expressed as N (%)

*HCL* Hospices Civils de Lyon; *yrs* years

### Dictionary of themes

The decomposition of the 10 061 comments into words and expressions by the linguistic analysis software gave 31 878 words and expressions. The exploratory thematic analysis without a priori found 28 general themes and 184 specific sub-themes (Appendices 1 and 2).

### Creation and analysis of the patient journey

The patient journey resulted in 26 themes distributed into 145 sub-themes. Two themes and 39 sub-themes were excluded, the reasons for exclusion are detailed in appendix 3. Among the 145 sub-themes, 124 were stated positively and negatively by the respondents and 21 were only stated negatively. The 5 most commented themes were: the perception of staff, their relational behaviours with the patient (*n* = 72%); the bedroom commodities (*n* = 30.6%); communication and information (*n* = 24.1%); catering (*n* = 23.2%); environment (*n* = 21.5%). The complete patient journey with the indicators for each theme and sub-theme is shared in appendix 4.

### Prioritisation of sub-themes

Among the 145 sub-themes, 23 fitted the prioritisation criteria established: four strengths, eight priorities, three good practices, and eight weak signals. They were then gathered into four key categories: relationship between patient and staff (Table 2), environment (Table 3), surgery and pain management (Table 4), and information and care coordination (Table 5).

**Table 2:** Relationship between patient and staff: characteristics of the sub-themes identified in the patient journey after prioritisation and actions proposed based on the comments in the category

Prioritisation	Description of the sub-theme	Content of the comments	Percentage of respondents and their characteristics	Actions / practices / expectations identified in the comments
Strengths	Quality of reception	Patients described a "*warm*", "*smiling*" and "*caring*" welcome	*n* = 10.3% All profiles	Importance of smile
	Perception of staff as being professional and competent	Perception of professionalism based on listening, follow-up, and reactivity. Better perception if there is an interaction with the surgeons and/or professors beyond care	*n* = 22.6% All profiles	Importance of the visit of the surgeon and/or professor
	Listening and availability	Patients described that it was not a question of the amount of time spent with the staff but rather their ability to be “*active listeners*” and to identify the times when they needed to be listened to (night, day without visitors, etc.)	*n* = 35.5% All profiles	Work on key moments when the patient most needs attention (nights, days without visitors, etc.)
	Kindness	This quality is expressed by the staff being in good mood (smiles, sense of humour, kind words)	*n* = 26.6% All profiles	Importance of smile and sense of humour
Priorities	Consideration of the patients	Behaviour failures: discussions in front of the patient; “*rushed”* visits; too many professionals during visits; no respect for privacy and intimacy	*n* = 8.6% Patients hospitalised in surgery departments (*n* = 76.5%)	Be careful regarding discussions between professionals (even in the corridors). Avoid too many professionals during visits and/or inform the patient in advance of the number of people present
	Patient support	Patients felt the wait for help was too long, that they must ask for help (lack of proactivity). Patients felt obliged to justify their request to get support	*n* = 7.1% Paediatric, maternity, and geriatric departments	Try not to question the request for help. Propose key moments of support: information meeting, pre-discharge briefing, etc
	Ability to reassure the patient	Patients described feelings of anxiety that arose during hospitalisation. These feelings were caused by a lack of information and waiting times. Comments described very specific moments where the patients and/or their relatives need to be reassured (waiting times with young children, diagnostic announcements, etc.)	*n* = 8.3% All profiles	Work on the key moments that trigger anxiety (diagnostic announcement, management of the relatives in paediatric departments)
Good practice	Patience of the staff	Staff managed to not show their constraints and remained available in all situations (elderly patients, foreigners, etc.) and context (work overload, emergency, etc.)	*n* = 0.9% All profiles	Try not to rush the patient even if the situation is tense
Weak signal	Lack of frankness, dishonesty on the part of the staff	Situations where respondents felt that they are “*not told everything*” about their health condition or the surgery they have undergone	*n* = 0.2% Patients hospitalised in surgery departments (*n* = 85.7%)	Ask more frequently if all the questions have been raised, if there are still doubts, questions

**Table 3:** Environment: characteristics of the sub-themes identified in the patient journey after prioritisation and actions proposed based on the comments in the category

Prioritisation	Description of the sub-theme	Content of the comments	Percentage of respondents and their characteristics	Actions / practices / expectations identified in the comments
Priorities	Satisfaction about the bedroom	Patients expressed dissatisfaction about shared bedrooms, they mentioned the size of the room, the quality of the furniture, the lack of adaptation to certain patients (disabled, obese, geriatric, etc.), the fact that they had to share a room with strangers. The patient experience was significantly better when patients were in a single room	*n* = 13% All profiles Higher dissatisfaction among patients hospitalised more than 4 days (successive moves, lack of knowledge of different neighbours, etc.) and among patients in paediatric/maternity departments (issues with the relatives)	Try not to move a patient several times during his hospitalisation Focus on solutions in paediatric/maternity departments given the presence of family members/relatives
	Shared room when patients had asked for a single one	Patients were surprised to find themselves in a shared room when a request for a single room had been made. Patients did not always understand why they “*ended up*” in a shared room	*n* = 7.7% All profiles	Better inform in advance the situation regarding the availability of rooms
Good practice	Atmosphere	This sub-theme was very dependent on the staff’s behaviours towards the patients (smile, politeness, etc.) and on the perception of the behaviours between professionals (greeting, respect for each other, no depreciation, etc.)	*n* = 1.2% Patients hospitalised in surgery departments (*n* = 84.6%)	Pay particular attention towards having a respectful attitude between professionals
Weak signals	Orientation	Difficulty in finding their way around the hospital	*n* = 0.7% Mentioned by patients hospitalised in one of the institutions of the HCL	Improvements needed: signs from public transport; indications to find the admission building; bigger signs (to increase visibility) and better placed. Patients asked to include them in the process of rethinking the signposting
	Smells	Patients pointed out that the water and the products used to clean the floor smell particularly “*bad”*	*n* = 1% Mentioned by patients hospitalised in one of the institutions of the HCL	Change the cleaning products
	Personal hygiene products	Lack of hygiene products (soap, shampoo, toothpaste)	*n* = 0.3% All profiles	Better inform/remind patients before hospitalisation of products needed. Offer emergency products
	Telephone	The telephone service was criticised by patients and their relatives for being too expensive and not working properly	*n* = 0.6% Elderly patients and their relatives	When elderly patients: increase vigilance on the phone service quality

**Table 4:** Surgery and pain management: characteristics of the sub-themes identified in the patient journey after prioritisation and actions proposed based on the comments in the category

Prioritisation	Description of the sub-theme	Content of the comments	Percentage of respondents and their characteristics	Actions / practices / expectations identified in the comments
Priorities	Perception of the quality of the surgery	Patients mentioned difficulties in assessing the quality of the procedure they had undergone. To assess it, they mentioned other elements (absence of pain, complications; relational behaviours among the staff; reputation of the institution and of the surgeons). The perception of quality no longer depended solely on the level of competence but relied above all on the information given. When there was a lack of information/communication felt by the patient, they thought there was a problem during the surgery	*n* = 8.2% Surgery and obstetric departments	Particular attention needed concerning communication before (to reassure, defuse anxieties, etc.), during (explanations from the anaesthetist, etc.), and after the surgery (details of the surgery, follow-up, etc.)
	Pain management	Two very significant negative subjects: a lack of responsiveness to pain and a pain that was not “*taken seriously”*, for which the patient had to “*prove”* his or her pain	*n* = 7.5% All profiles	Try not to question the pain expressed by the patient
Good practice	Care during surgery preparation	Surgery preparation: information/ explanations, relational behaviours, heated operating table, etc	*n* = 1.6% Surgery department	Each member of the staff should introduce him/herself (first name)

**Table 5:** Information and care coordination: characteristics of the sub-themes identified in the patient journey after prioritisation and actions proposed based on the comments in the category

Prioritisation	Description of the sub-theme	Content of the comments	Percentage of respondents and their characteristics	Actions / practices / expectations identified in the comments
Priority	Communication and access to information	Patients regretted a lack of information, information that was not “*spontaneous*” and for which the patient must “*dig deeper*” (make several requests), information that was not very precise or too precise, staff who did not adapt to the patient, badly chosen “*information moments*”	*n* = 6.8% All profiles. Higher dissatisfaction among women in maternity departments	Proactivity in giving information Try to adapt the information to the patient Ask the patient if the moment is convenient
Weak signals	Coordination and communication between teams	Lack of communication between teams: the patient must inform the various people involved and repeat information. They also mentioned errors and missing information in their files	*n* = 1.5% All profiles	Ensure greater use of the patient record (or use a summary sheet with the most relevant information) Ensure identity surveillance
	Contradictory opinions	Patients described opinions that differed according to the professionals encountered. Two subjects stood out: organisation regarding hospital discharge and the specific subject of breastfeeding	*n* = 0.8% All profiles. Higher dissatisfaction among women in maternity departments	Be careful to harmonise information, especially during staff turnover
	Birth registration service	Non-efficient process for declaring stillbirths	*n* = 0.2% Women in maternity departments	Try to facilitate/support the process for declaring children born without life

Within the first category, the perception of professionals was the most commented theme (*n*=72%) and contained the greatest positive speech engagement; 8/14 sub-themes had a positive mean satisfaction. The four strengths identified in this category were easily shared with the staff, as they described the positive perceptions of the patients regarding staff behaviour and highlighted simple actions/practices that greatly impact PE (e.g., importance of smile and sense of humour: “*nurses, always with a smile and a touch of humour to make you think of other things*”). One strength revealed that patients are aware of the staff’s working conditions and thus do not expect them to be constantly available. Instead, they valued the capacity of the staff to be available and active listeners in key moments (e.g., night, days without visitors). The priorities and week signal identified in this category showed a need for improvement on three main subjects, specifically in the surgery department: raise staff awareness regarding their discussions in front of the patients or in the corridors, limit or inform the patient of the number of professionals that will be present during the visit (academic hospital), ask more frequently (especially during the visit) if the patient has questions. In paediatric, maternity, and geriatric departments, the patients shared a negative perception about a lack of support from the staff. They had the feeling that they must ask for help and felt obliged to justify their request to get support. To illustrate this point, a woman commented: “*I was exhausted, and I decided to leave my baby in the nursery. Two nurses asked me why, I replied that I was very tired and that it was the first time I had asked for help* […] *I had a hard time with this because I had to justify myself and then feel guilty for leaving her*”. To deepen this perception, we also investigated the feeling of a lack of reassurance that illustrates the need for patients to be better informed and accompanied by the staff, particularly during stressful moments (e.g., waiting times with young children, diagnostic announcements; Table 2).

Regarding the second category, the perceptions of patients were negatively impacted by the dissatisfaction about shared bedrooms (*n*=13%). This was particularly relevant in the paediatric and maternity departments, where the need to manage the discomfort of the patient and the relatives was largely commented on. This dissatisfaction also came from the misunderstanding of the patients who had asked for a single room but found themselves in a shared room (*n*=7.7%). The weak signals of this category allowed to further identify specific expectations regarding the hospital environment, and two of them were specific to one of the institutions of the HCL. For instance, an orientation issue (*n*=0.7%) was described by patients having trouble finding their way in the institution. The comments proposed to include patients in the process of rethinking the orientation panels and clearly stated which aspects needed improvement, e.g.: “*signs orienting the places* […] *are too small* […] *poorly signposted and badly placed, not highlighted (sign background standing out from the environment)*” (Table 3).

The negative perceptions concerning surgery and pain management were not caused by a high level of pain but rather by moments where the patient felt his/her pain was not “*taken seriously*” by the staff or that he/she “*must prove*” his/her pain. This category also highlighted the discrepancy between evidence-based results (e.g., success of the surgery) and PE. For example, the priority “*perception of the quality of the surgery*” described patients seeking to assess the quality of their surgery. In this situation, patients relied on what they felt (absence of pain) and what was communicated to them before (to reassure, defuse anxieties, etc.), during (explanation of the gestures, etc.), and after the surgery (details of the surgery, follow-up, etc.). The comments described a good level of information before (preparation for the surgery was a good practice) and during the surgery, but were negative regarding communication after the surgery, e.g.: “*little information on the surgery itself, except a "it went well", you must beg for details”.* The lack of information after the surgery triggered a feeling of mistrust from the patient: "*if they don't tell me anything, it means there is something to hide*" (Table 4).

The last category emphasized the lack of information (*n*=6.8%) received by patients who explained they must “*dig deeper*” to obtain detailed information. Some patients (*n*=1.5%) explained this by a bad coordination between teams, describing reverse situations where the patient would inform the staff about their situation. However, contrary to other departments, the women in maternity departments regretted an excessive amount of information, e.g.: “*I was overwhelmed with information which put me under a lot of stress for fear of forgetting something*”. Furthermore, a weak signal (*n*=0.8%) described conflicting advice about breastfeeding: “*too many professionals with differing opinions. In maternity, it is a bit confusing and stressful.*”. The last weak signal was rarely commented on (*n*=0.2%) as it only concerned stillbirths and the process for declaring children born without life (Table 5).

### Usefulness of the non-prioritised sub-themes from the patient journey

The sub-themes that were not prioritised were nevertheless used to help specific staff improve their quality of service. For instance, the staff in charge of the catering was made aware of the expectation of patients to have access to a greater variety of menus. Similarly, the staff in charge of improving the care of patients with disabilities was told that patients describing themselves as deaf asked to use a light signal in certain rooms rather than a knock on the door.

## Discussion

Over a year, nearly 80% of the respondents to the French PE survey left a comment after their hospitalisation in the HCL. The analysis of the surveys containing comments allowed to create a patient journey with a very high number of precise sub-themes. More than 15% of these were prioritised using the present methodology and are described in operational sheets to help institutions, departments, and staff conduct quality improvements regarding four key categories: relationship between patient and staff; environment; surgery and pain management; information and care coordination.

The results obtained herein reflect those found in the existing literature, particularly the fact that more than half of the themes mentioned in patient comments were not present in the survey’s closed-ended questions [16] and that patient narratives can improve healthcare quality beyond what standardised survey scores can accomplish [10, 17]. The most commented themes and sub-themes identified herein also confirm those reported in the literature, especially the importance of patient-staff interaction to improve patient journey [18], and the need to improve information in the aftercare [19].

The four key categories identified herein are in line with the primary dimensions of health service quality -interpersonal, technical, environment, and administrative quality-, as described by Dagger et al. (2007) [20]. The relationship between patient and staff is predominantly determined by the interpersonal quality of care, which reflects the ability of healthcare providers to establish and maintain relationships with patients. The environment in which care is delivered encompasses non-medical aspects of care such as accessibility, comfort, and the overall care experience. Surgery and pain management are primarily linked to the technical quality, which encompasses the clinical skills and expertise of healthcare providers. Dagger's model also includes an administrative dimension, which reflects the efficiency and effectiveness of the healthcare system as a whole. This dimension is closely related to the information and care coordination aspect of PE identified herein. The administrative dimension encompasses factors such as waiting times, errors in patient files, and internal processes, which are dependent on effective information and communication systems. Therefore, ensuring effective information and communication is necessary for achieving high levels of administrative quality, which in turn contributes to the overall quality of care provided to patients.

Some expectations reported by the patients are specific to the present analysis as they concern the specific institution commented on (for example, the orientation issues) or are specific to French hospitals (for example, the incomprehension of patients who find themselves in a shared room despite their request for a single one and despite the fact that they pay their insurance company to cover this particular service).This approach places the patient's view at the heart of the institutional strategy while combining it with action levers that are directed at the department and staff level. It also encourages the engagement of patients by bringing patient comments to light. Communication of the results and the actions defined should also increase the overall engagement of patients in the process. Identifying such actions at different levels and on different time scales allows to rapidly implement targeted actions at the department and staff level while developing long-term institutional strategies, thus enabling to keep a balance between the improvements needed and the daily functioning of an academic hospital federation. For example, a first step consisted in valuing staff for their work by sharing the practices commented on by patients and the good quantitative and qualitative feedbacks. To this end, the modelling of the patient journey represented a good pedagogical tool when sharing the results to the staff. Another type of rapid action concerns practical improvements (e.g., orientation issues, information before admission, birth registration process) implemented by support teams, thus avoiding the burdening of front-line professionals. In a second and longer step, improvements such as the need to improve aftercare information, became part of the institutional strategy which aimed to engage staff at different moments of the patient journey (e.g.: after care, during preparation for discharge, follow-up after discharge, etc.). Some of the improvements needed (such as pain management and behaviours during visits) are not limited to the institutions included as such issues can be addressed during the initial training of the professionals (schools or universities). In this specific context of an academic hospital federation, the results will be shared with the actors involved in initial training with the aim to raise awareness on patients’ expectations as early as possible.

The methodology applied herein is particularly relevant to reflect PE and conduct changes at the staff, department, and institution level. For instance, another analysis conducted on the same material but using a different methodology (word and comment filtering and non-negative matrix factorisation algorithm), allowed to identify about twice as less themes compared to the present methodology [21]. That specific analysis, conducted by the French National Authority for Health (*Haute Autorité de Santé*, HAS), differed however, as it aimed to identify frequently raised themes and included more than two million comments. Another difference relates to the choice to analyse themes and sub-themes commonly, whether they are mentioned in the positive or negative question. This approach, which was applied herein, allows to measure how often a theme or a sub-theme is mentioned (positively or negatively), to determine the mean overall satisfaction regarding the theme/sub-theme, and to measure the proportion of positive and negative speech. This therefore allows to identify the reasons of dissatisfaction but also the practices valued by the patients on a same theme. Furthermore, when sharing the results with the staff, it contributes to conveying the results in a positive and engaging manner. The constraint of a joint approach, however, lies in the additional analytical time required, as it is necessary to differentiate positive, neutral, and negative speech. The present analysis also proposes to consider the syntax of the comments, allowing to measure speech engagement [14], to go beyond the mere recurrence of themes in order to prioritise improvement actions, and to highlight sub-themes that are rarely mentioned. In both approaches, the quantification of the identified themes allowed to transform qualitative material into measurable and comparable data that could allow comparisons in the long-term, given that the expression mode of patients remains stable over time. This expression mode depends on the case mix (age, sex, type of disease), the educational, and socio-economic level of patients [22, 23]. The selection of an analytical method must thus consider the number of respondents, the expected outcomes, the time constraints, and the technical challenges. A manual analysis, which can be time consuming, enables to overcome certain challenges met by natural language processing (NLP) as, according to Greaves et al., “*machines struggle to read and understand comments accurately; software finds comments preceded by negatives difficult to interpret*”^9^. Furthermore, the use of sarcasm and irony – a feature of British and French cultures- is hard to process by machines [9]. The proposed approach enables to analyse many patient comments, allowing a comparability of results between institutions and over time, which is difficult to obtain on small samples using methods such as qualitative interviews or focus groups.

The present study has certain limitations. The use of comments from open-ended questions that are placed after closed-ended questions may bias the content of the comments. However, one study tested the variation in narrative content and quantitative scores as a function of the placement of open-ended questions on the Consumer Assessment of Healthcare Providers and Systems (CAHPS) survey. The results showed that the relative placement of closed and open-ended questions had little impact on CAHPS narratives or scores [24]. The use of the e-Satis survey implies a bias in the selection of respondents, as it implies needing an e-mail address, answering the online survey, etc. This bias, however, is limited by the manual intervention which enables to identify specific sub-themes raised by only a few respondents and the use of patient characteristics that allows to target specific populations. An important limitation when analysing patient comments in healthcare setting is the time and financial cost that can be incurred. In the present research framework, which was part of a public health and management science thesis, no financial or time constraints were encountered. A budget and time estimate however, were simulated for the entire process, including researcher compensation, software usage, document production, and other expenses. A mean time of six weeks and a cost of 25,000 euros were estimated. The number of comments analysed is the most critical variable in determining costs. The present research protocol involved analysing all the comments over a specific period, but sampling techniques could be used to adjust analysis costs. Ultimately, the use of patient experience data from the years 2018–2019 may be subject to limitations in light of the COVID-19 pandemic. It is possible that patients' perceptions of healthcare quality and their overall experience of care may have changed as a result of the pandemic. For instance, concerns about infection control and access to care may have become more salient in patients' minds, potentially impacting their satisfaction regarding healthcare services. Similarly, changes to healthcare delivery models and resource allocation may have influenced patients' experiences of care in ways that are not captured in the data from 2018–2019. As such, it is important to interpret the results of the present study with caution and consider the potential impact of COVID-19 on patients’ perceptions of healthcare quality. Future research may need to incorporate more recent data to capture the evolving nature of patient experience in the context of the pandemic.

The next step in our research will consist in involving the staff to collect and analyse their perceptions regarding their interactions with patients in order to balance the present patient-centred results. This would allow to refine improvement actions to remain as close as possible to the staffs’ specific needs and engage staff in a dynamic process for improving their practice and patient care.

## Conclusions

The analysis of patient comments obtained from a standardised survey allowed to characterise the patient journey using data that describes PE, further enabling a prioritisation of actions aiming to improve practice and quality of care at the institution, department, and staff level.

## Key points for decision makers

- Although measurement of patient experience using surveys has greatly been developed in the recent years, the effective use of the data obtained to improve practice and quality of care remains debated.
- In an institutional strategy to improve patient experience, a thematic and syntactic analysis of comments from patient experience national surveys allows to identify a great variety of specific themes and sub-themes. This enables to identify and categorise different levels of actions according to different timeframes (quick operational actions and long-term improvements) that involve different members of staff (e.g., nurses, physicians, administrative teams).
- Translating qualitative data obtained from patient comments into quantitative indicators allows to create a patient journey of which each step can be analysed and prioritised to implement field-oriented actions to improve quality of care and help staff improve their practice.

### Supplementary Information

**Additional file 1:** **Appendix 1.** Example of themes and sub-themes with their positive and negative qualification, taken from the e-Satis verbatims of the HCL. **Appendix 2.** List of the 28 general themes and 184 specific sub-themes from the HCL e-Satis verbatims. **Appendix 3.** Reasons for exclusion of the 2 themes and 39 sub-themes from the patient journey, based on the list of the 28 general themes and 184 specific sub-themes from the HCL e-Satis verbatims. 

**Additional file 2:** **Appendice.**

## Data Availability

Details of the data used and analysed are shared in the appendix. The exhaustive datasets used and/or analysed during the current study are available from the corresponding author on reasonable request.

## Abbreviations

ATIH: Agence Technique de l’Information sur l’Hospitalisation (French technical agency for information on hospitalisation)
CAHPS: Consumer Assessment of Healthcare Providers and Systems
CNIL: Commission Nationale de l’Informatique et des Libertés (French National Authority for Data Protection)
HAS: Haute Autorité de Santé (French National Authority for Health)
HCL: Hospices Civils de Lyon
MCO: Médecine, Chirurgie, Obstétrique (medical, surgery, obstetric)
NLP: Natural language processing
PE: Patient Experience
