# Supplementary material for: Using patient comments from a standardised experience survey to investigate their perceptions and prioritise improvement actions: a thematic and syntactic analysis
Source: BMC Health Serv Res. 2023 Sep 14;23:988. doi: 10.1186/s12913-023-09953-z (PMC10503051; doi:10.1186/s12913-023-09953-z)

PATIENT EXPERIENCE JOURNEY

HCL
HOSPICES CIVILS DE LYON

Patient journey
based on the analysis
of e-Satis verbatims of the HCL

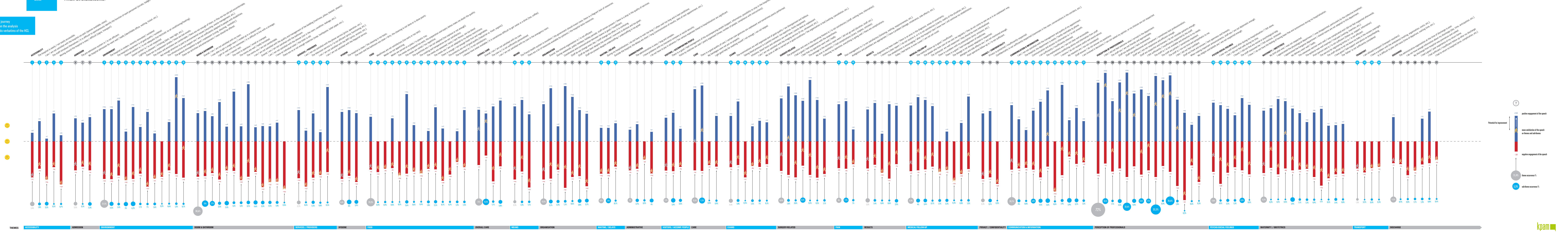

Supplement: Supplementary file 2 — Additional file 2: Appendice. [file 12913_2023_9953_MOESM2_ESM.pdf]
