# Supplementary material for: Using patient comments from a standardised experience survey to investigate their perceptions and prioritise improvement actions: a thematic and syntactic analysis
Source: BMC Health Serv Res. 2023 Sep 14;23:988. doi: 10.1186/s12913-023-09953-z (PMC10503051; doi:10.1186/s12913-023-09953-z)
Supplement: Supplementary file 1 — Additional file 1: Appendix 1. Example of themes and sub-themes with their positive and negative qualification, taken from the e-Satis verbatims of the HCL. Appendix 2. List of the 28 general themes and 184 specific sub-themes from the HCL e-Satis verbatims. Appendix 3. Reasons for exclusion of the 2 themes and 39 sub-themes from the patient journey, based on the list of the 28 general themes and 184 specific sub-themes from the HCL e-Satis verbatims. [file 12913_2023_9953_MOESM1_ESM.pdf]

APPENDICES

Appendix 1. Example of themes and sub-themes with their positive and negative qualification, taken from the e-Satis verbatims of the HCL.

**THEME: Catering**				**THEME: Perception of staff**			
**Sub-theme: cooking / temperature**		**Sub-theme: variety**		**Sub-theme: approachable**		**Sub-theme: patience**	
Words and expressions included	Positive (1) or negative (2) qualification	Words and expressions included	Positive (1) or negative (2) qualification	Words and expressions included	Positive (1) or negative (2) qualification	Words and expressions included	Positive (1) or negative (2) qualification
bonne cuisson vapeur des légumes	1	*manque de variété des menus*	2	*accessibilité du chirurgien*	1	*chirurgien a été patient*	1
méthode de cuisson à revoir	2	*nourriture n'est pas variée*	2	*interne très accessible*	1	*manque de patience cruelle*	2
pates souvent trop cuites	2	*repas plutôt bons et variés*	1	*personnels très avenants*	1	*secrétaires très patientes*	1
viandes trop cuites et dures	2	*3 fois des pates*	2	*équipes médicales très accessibles*	1	*une grande patience avec les patients*	1
(...)		(...)		(...)		(...)	

Appendix 2. List of the 28 general themes and 184 specific sub-themes from the HCL e-Satis verbatims.

Theme	N°	Sub-theme	Theme	N°	Sub-theme
**1. Accessibility / identification**	1	Accessibility	**Maternity**	93	Nursery
	2	Orientation		94	Physiological
	3	Parking		95	Post-partum
	4	Staff identification		96	Choices respected
**2. Administrative**	5	Patient file	**15. Medical care**	97	Personalised medicine
	6	Rates and payments		98	Care response to emergencies
	7	Social security		99	Dosage
**3. Bedroom and bathroom**	8	Furnishing	**16. Medical monitoring**	100	Medical monitoring
	9	Bathroom general condition		101	Medical follow-up
	10	Bedroom general condition		102	Medications
	11	Comfort		103	Postoperative follow-up
	12	Neighbour		104	Prescription
	13	Quality of the bed		105	Protocols
	14	Shower		106	Side effects
	15	Single or shared room		107	Staff visits
	16	Size of the room	**17. Medical results**	108	Reports
	17	Sleep and rest		109	Diagnostic
	18	Toilet		110	Health results
	19	Washing and toilet products		111	Rehabilitation
**4. Catering / Food service**	20	Appropriate to the patient's needs	**18. Nursing care**	112	Diaper
	21	Adapted quantities		113	Care procedures
	22	Appetizing		114	Dressing, bandage
	23	Breakfast		115	Patient hygiene
	24	Food service (general)	**19. Operative**	116	Anaesthesia
	25	Containers		117	Care before the surgery
	26	Expiry date		118	Postoperative
	27	Flavour		119	Recovery room
	28	Fruits, vegetables		120	Scars, stitches

	29	Mealtime		121	Operative
	30	Quality of the products	20. Other sub-themes	122	Acknowledgements of the patients
	31	Cooking / temperature		123	Body parts
	32	Variety		124	Clothing
	33	Water, drinks		125	Floor names
**5. Cleanliness**	34	Cleanliness of the environment		126	Generic terms for hospitalisation
	35	Cleanliness of the patient's room		127	Generic terms for institution
**6. Communication & information**	36	Patient agreement		128	Generic terms for patient
	37	Advice		129	Generic terms for treatment
	38	Answers provided to questions		130	Night
	39	Appointments		131	Paediatric department
	40	Clarity of explanations		132	Intensive care department
	41	Consideration of requests		133	Transfer
	42	Contradictory opinions	**21. Pain**	134	Pain killers
	43	General information		135	Pain management
	44	Sufficient information	**22. Parents and relatives**	136	Parents and relatives
	45	Phone contact		137	Visiting procedure
**7. Confidentiality and privacy**	46	Medical secrecy	**23. Public institution**	138	Perception of public hospital
	47	Respect of privacy		139	Spending
**8. Entry**	48	Admission	**24. Perception of staff**	140	Approachable
	49	Preadmission		141	Attention given during diagnosis announcement
**9. Environment**	50	Animations and entertainments		142	Behaviours and attitudes (general)
	51	Atmosphere		143	Honesty
	52	Chairs		144	Involvement
	53	Ecology		145	Kindness
	54	Equipment		146	Listening
	55	Equipment for handicap situation		147	Patience
	56	Infrastructure (general)		148	Professionalism
	57	Light, luminosity		149	Reactivity
	58	Odours		150	Respectful of religion
	59	Old or modern environment		151	Respect, consideration
	60	Sound comfort		152	Support
	61	Thermic comfort		153	Trust
**10. Examination**	62	Examination		154	Welcoming
	63	Blood pressure	**25. Private services**	155	Happytal
	64	Perfusion, blood test		156	Phone
	65	Radiograph, doppler		157	Tv
	66	Scanner		158	WIFI
**11. Exit**	67	Administrative process	**26. Staff**	159	Administrative
	68	Discharge (general)		160	Anaesthesiologist
	69	Discharge information		161	Care assistants
	70	Follow-up after discharge		162	Caregivers
	71	Must return to hospital		163	General practitioner (out of hospital)
	72	Patient feels that his stay is too short		164	Hospital porters
	73	Return home		165	Housekeeping staff
**12. Feelings**	74	Autonomy		166	Interns
	75	Being surrounded vs. loneliness		167	Medical staff
	76	Fears, worries, anxieties		168	Midwives
	77	Feeling of security or insecurity		169	Night team
	78	Psychological and social care		170	Nursery assistants
**13. Overall organisation**	79	Staff work overload		171	Nurses
	80	Organisation within the departments		172	Obstetricians and gynaecologists
	81	Schedule during specific moments: weekends, holidays, public holidays		173	Paramedical
	82	Staff coordination		174	Paediatrics
	83	Team rotation		175	Physical therapists
	84	Under-staffed		176	Physicians
**14. Maternity**	85	Delivery		177	Surgeons
	86	Breastfeeding		178	Teachers
	87	Caesarean		179	Undetermined staff
	88	Declaration of birth	**27. Transports**	180	Ambulance
	89	Epidural		181	Taxi
	90	Father / close companion		182	Transport vouchers
	91	Kangaroo department (fragile new-borns)	**28. Wait**	183	Waiting times
	92	Maternity (general)		184	Delays

Appendix 3. Reasons for exclusion of the 2 themes and 39 sub-themes from the patient journey, based on the list of the 28 general themes and 184 specific sub-themes from the HCL e-Satis verbatims.

Reason of exclusion	Use	Themes and sub-themes
Theme and sub-themes that identified the staff but did not correspond to a sub-theme of the patient journey (n=1 theme; n=21 sub-themes).	Used to cross-reference sub-themes. For example, to study how many times the subject of listening is mentioned specifically in relation to nurses.	**26. Staff (theme)**<br>159 Administrative<br>160 Anaesthesiologist<br>161 Care assistants<br>162 Caregivers<br>163 General practitioner (out of hospital)<br>164 Hospital porters<br>165 Housekeeping staff<br>166 Interns<br>167 Medical staff<br>168 Midwives<br>169 Night team<br>170 Nursery assistants<br>171 Nurses<br>172 Obstetricians and gynaecologists<br>173 Paramedical<br>174 Paediatrics<br>175 Physical therapists<br>176 Physicians<br>177 Surgeons<br>178 Teachers<br>179 Undetermined staff
Theme and sub-themes that did not present convincing	Not used.<br>(For example, the sub-theme “clothing” was	**20. Other (theme)**<br>112 Diaper<br>123 Body parts<br>124 Clothing<br>125 Floor names

quantitative and qualitative results (n=1; n=9 sub-themes).	commented on by 0.3% of respondents with very low engagement).	126 Generic terms for hospitalisation<br>127 Generic terms for institution<br>128 Generic terms for patient<br>129 Generic terms for treatment<br>130 Night
Generic sub-themes with very short words or expressions (1 or 2 words; n=5 sub-themes).	Included for the generation of statistics of the theme (for instance when a patient just responded: "*food*").	24 Food service (general)<br>56 Infrastructure (general)<br>68 Discharge (general)<br>92 Maternity (general)<br>142 Behaviours and attitudes (general)
Sub-themes redundant between quantitative and qualitative data (n=3 sub-themes).	These 3 subjects were already identified in the quantitative data (for example, the information of the survey came from the paediatric department or elsewhere was already known).	131 Paediatric department<br>132 Intensive care department<br>133 Transfer
Sub-theme that did not correspond to a sub-theme of the patient journey (n=1 sub-theme).	Used for HCL internal communication and for further research considering staff perceptions.	122 Acknowledgements of the patients
